# Supplementary material for: Real-time tracking of complex ubiquitination cascades using a fluorescent confocal on-bead assay
Source: BMC Biol. 2018 Aug 10;16:88. doi: 10.1186/s12915-018-0554-z (PMC6086040; doi:10.1186/s12915-018-0554-z)
Supplement: Supplementary file 1 — Contains Supplementary Figures S1-S7. Figure S1. Ring fluorescence intensity is linearly proportional to the amount of bead-bound fluorescent substrate and is consistent across a test plate. Figure S2. On-bead ubiquitination of a protein substrate, p53. Figure S3. Fluorescence excitation and emission spectra for simultaneous detection of three different fluorescent-fusion proteins. Figure S4. Bead detection and analysis allows for differential detection of mixed small and large bead populations. Figure S5. Inhibitory effects observed in time-resolved ubiquitination cascade reactions. Figure S6. Concentration-dependent inhibitory activity of BAY 11-7082 observed on eGFP-Ube2L3 using UPS-CONA. Figure S7. Ring fluorescence intensity is stable over time and remains linearly proportional to the amount of bead-bound fluorescent substrate. Table S1. UPS-CONA is a broadly applicable assay. (DOCX 6279 kb) [file 12915_2018_554_MOESM1_ESM.docx]

Additional File 1

**Real-time tracking of complex ubiquitination cascades using a fluorescent confocal on-bead assay**

Joanna Koszela*, Nhan T Pham, David Evans, Stefan Mann, Irene Perez-Pi, Steven Shave, Derek F J Ceccarelli, Frank Sicheri, Mike Tyers, Manfred Auer*

**Supplementary Figures**

**
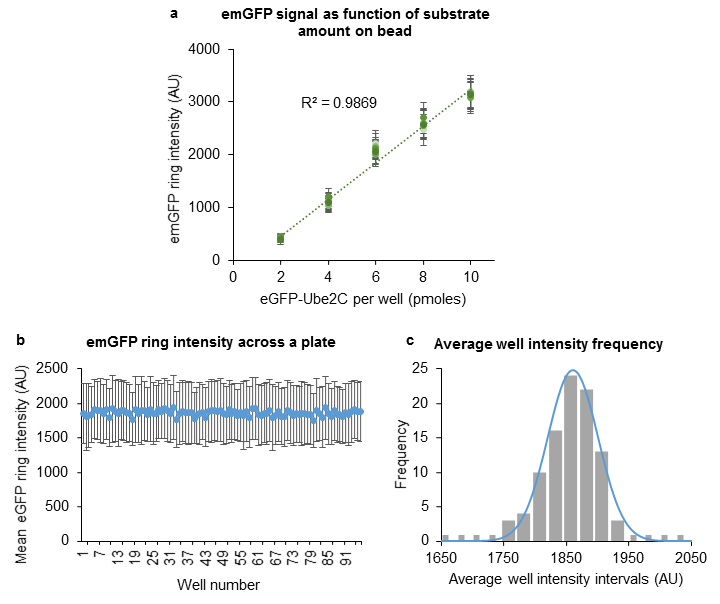
**

**Figure S1: Ring fluorescence intensity is linearly proportional to the amount of bead-bound fluorescent substrate and is consistent across a test plate.**

(**a**) His6-tagged emGFP-Ube2C fusion in different amounts (0-10 pmoles per well) was conjugated to Ni^2+^NTA agarose beads as described in the Methods. After washes, the emGFP-Ube2C beads were placed in a 384-well plate and imaged using the confocal scanning microscope Opera^TM^ (Perkin Elmer). The ring emGFP fluorescence intensity was averaged for each well. Detected emGFP intensity increased linearly to the expected amount of the bead-bound emGFP-Ube2C. (**b**) A batch of His6-tagged emGFP beads was prepared at a concentration of 250 nM (5 pmoles per well) and distributed into 96 wells across a 384-well plate. Images were acquired on the Opera^TM^ (Perkin Elmer). The detected emGFP intensity is consistent across the plate. (**c**) Frequency of wells with a given mean of emGFP intensity follows a normal distribution.

**
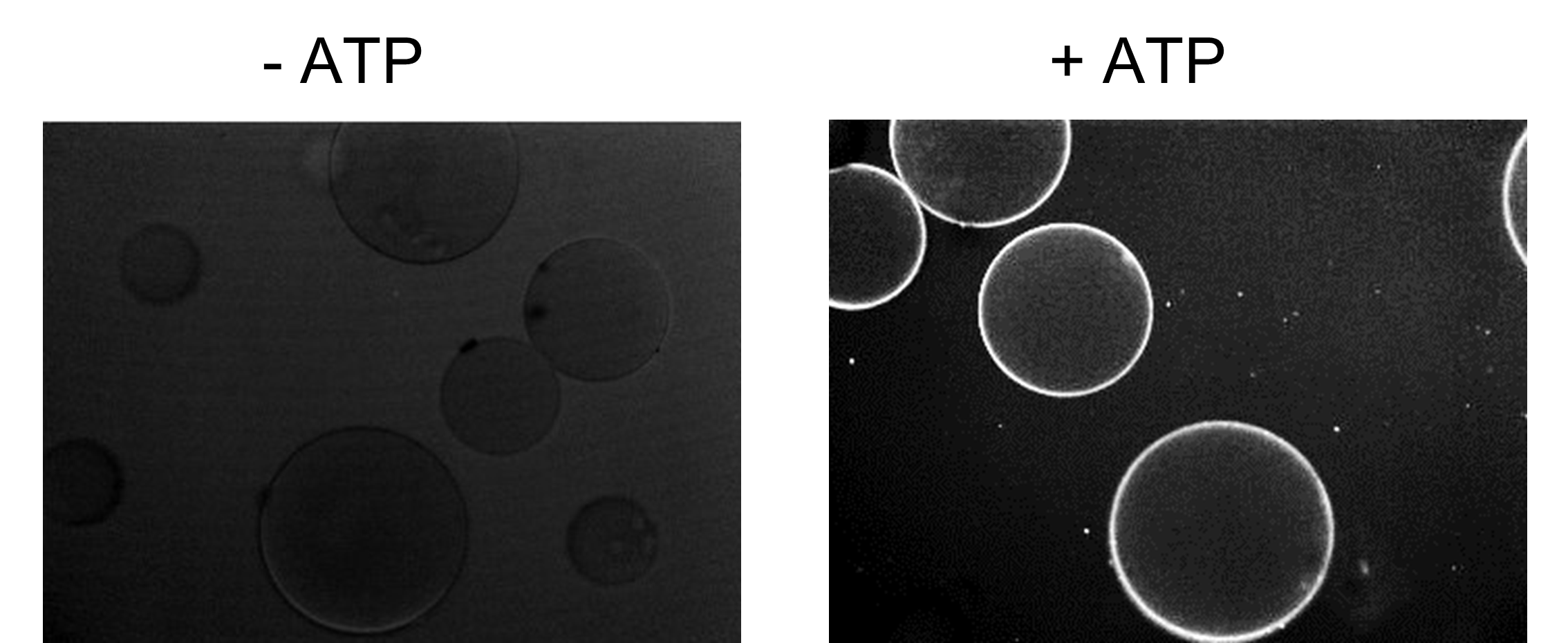
**

**Figure S2: On-bead ubiquitination of a protein substrate, p53.**

The tumour suppressor protein p53 tagged with His6 on the N-terminus (BostonBiochem, cat. no SP-40) was immobilized on Ni^2+^NTA agarose beads as described in the Methods. 150 pmoles of p53 was used per well. Washed p53 beads were placed in a 384-well plate and incubated with 500 nM FITC-Ub (LifeTechnologies, cat. no PV4378), 100 nM Ube1 (BPS Bioscience, cat. no 80301), 2.5 µM Ube2D2 (BostonBiochem, cat. no E2-622), 84 nM Mdm2 (Millipore, cat. no 23-032) in energy-regeneration buffer (50 mM Tris-HCl, pH 7.5, 5 mM MgCl_2_, 10 mM creatine phosphate, 3.5 U/mL creatine phosphokinase), in the absence or presence of ATP. After 6 h incubation at room temperature, the imaged were acquired on the Opera^TM^ (Perkin Elmer) using FITC detection settings and the images were processed using ImageJ software. Bright rings appear upon addition of ATP, corresponding to FITC-Ub conjugation to the on-bead p53.


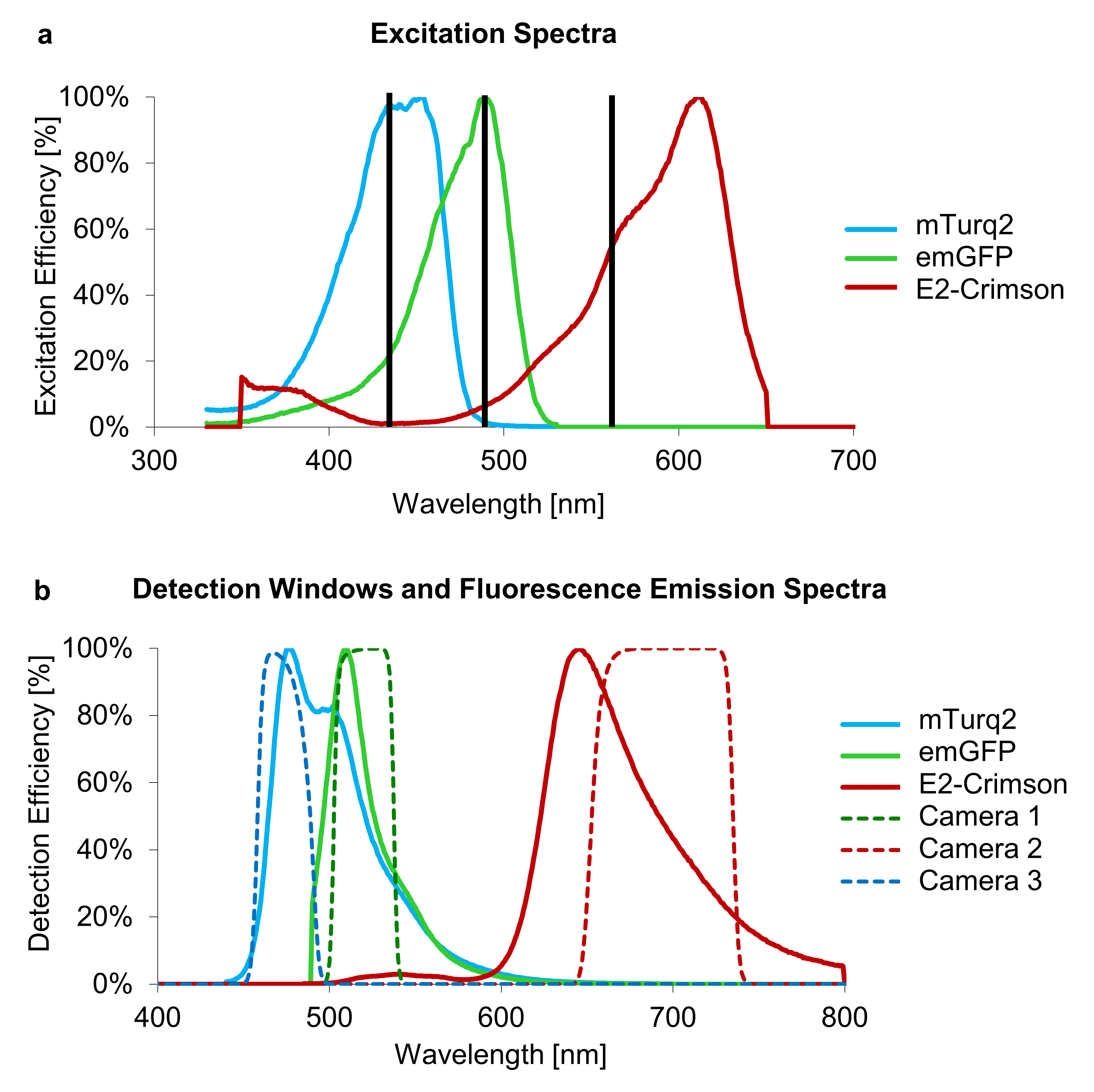


**Figure S3: Fluorescence excitation and emission spectra for simultaneous detection of three different fluorescent-fusion proteins.**

(**a**) Excitation spectra for mTurquise2 (mTurq2, blue), emGFP (green) and E2-Crimson (red) are represented, together with excitation wavelengths (black spikes) used on the confocal fluorescence scanning microscope Opera^TM^ (Perkin Elmer) for excitation of these proteins: 445, 488 and 561 nm, respectively. (**b**) Emission spectra of mTurq2, emGFP and E2-Crimson were overlaid with corresponding emission filters on the Opera^TM^ (Perkin Elmer) used for detection of these proteins: Camera 3: 475/34 nm, Camera 1: 520/35 nm and Camera 2: 660/150 nm.

*
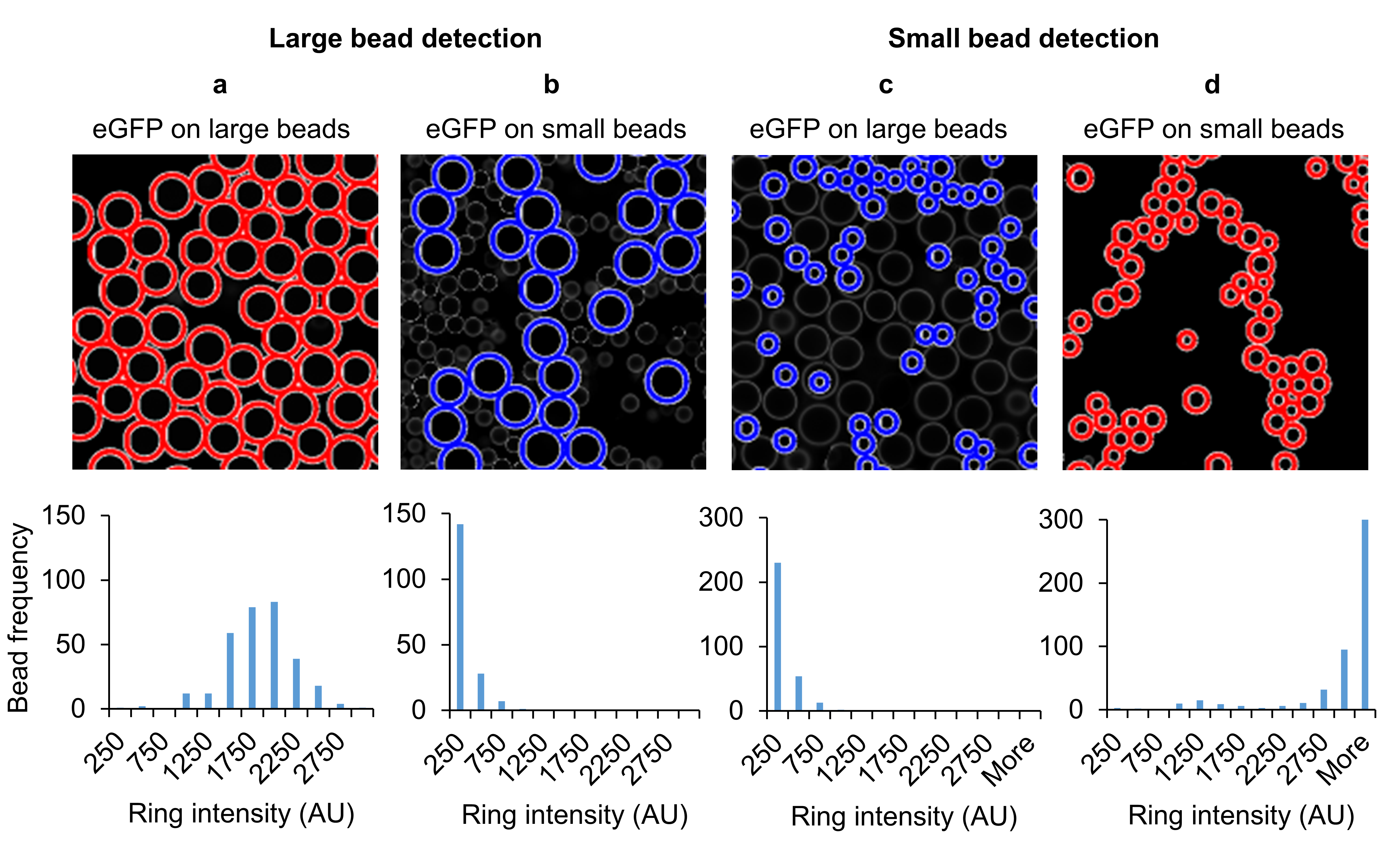
*

**Figure S4: Bead detection and analysis allows for differential detection of mixed small and large bead populations.**

emGFP was immobilised on small (40-70 µm) or large (100-120 µm) beads as described in the Methods and mixed with large or small (respectively) blank beads in the same well, resulting in a mixed bead populations: fluorescent large beads and blank small beads (**a** and **c**) or blank large beads and fluorescent small beads (**b** and **d**). Images were acquired in the brightfield and GFP detection channels using the Opera^TM^ microscope (Perkin Elmer) and submitted to bead detection and analysis as described in Methods. When the parameters were set for detection of large beads (**a** and **b**), large beads were detected, either from the GFP detection image (GFP on large beads, red circles) or from the brightfield image (large blank beads, blue circles). Similarly, when the parameters were set for detection of small beads (**a** and **b**), small beads were detected from the GFP detection image (GFP on small beads, red circles) or from the brightfield image (small blank beads, blue circles). Distribution of detected bead intensity corresponding to each well and to detection settings as indicated is represented in the charts: high GFP intensity for GFP on large beads, large beads detection **(a)** and for GFP on small beads, small bead detection **(d)**; null to low GFP intensity for GFP on small beads, large beads detection **(b)** and for GFP on large beads, small beads detection **(c)**.


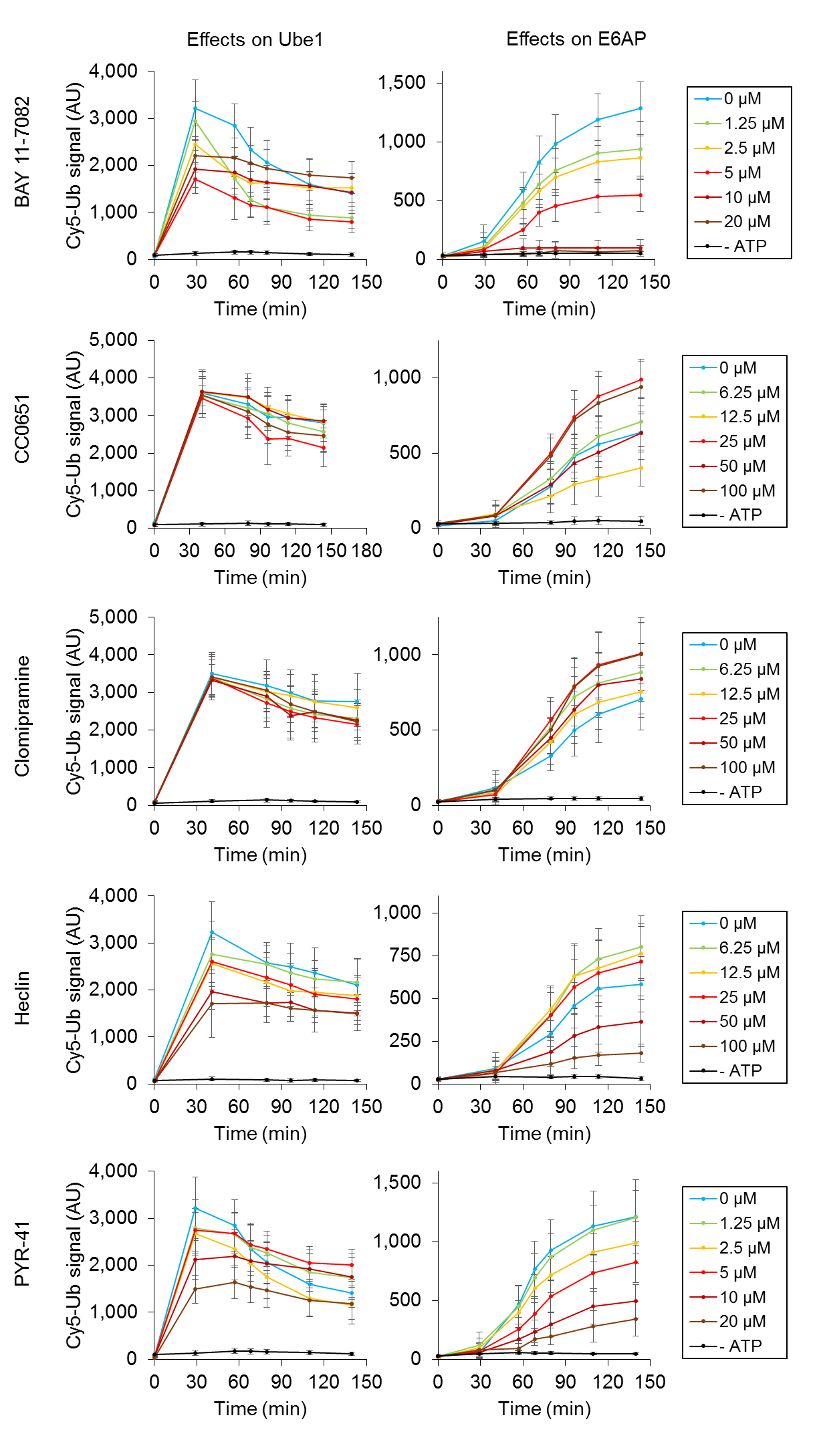


**Figure S5: Inhibitory effects observed in time-resolved ubiquitination cascade reactions.**

Reactions were prepared as detailed in the Methods in the presence of five ubiquitination inhibitors as indicated and ubiquitin charging to Ube1 and conjugation to E6AP was monitored using UPS-CONA.


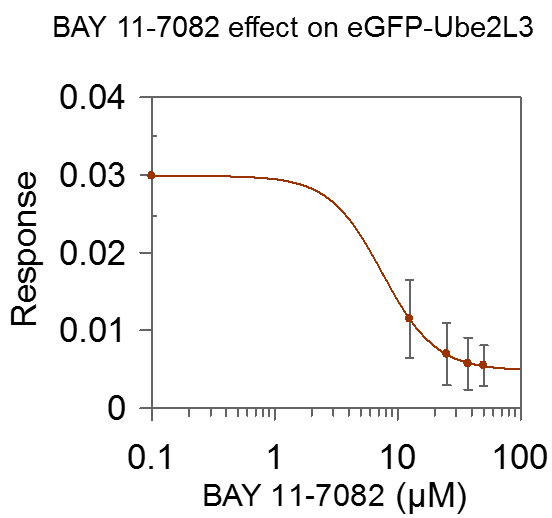


**Figure S6: Concentration-dependent inhibitory activity of BAY 11-7082 observed on eGFP-Ube2L3 using UPS-CONA.**

eGFP-Ube2L3 was immobilized on beads, preincubated with BAY 11-7082 at indicated concentrations and subjected to ubiquitination assay as detailed in Methods. Data points were fitted to a four-parameter logistic IC50 function and represented using GraFit7.

*
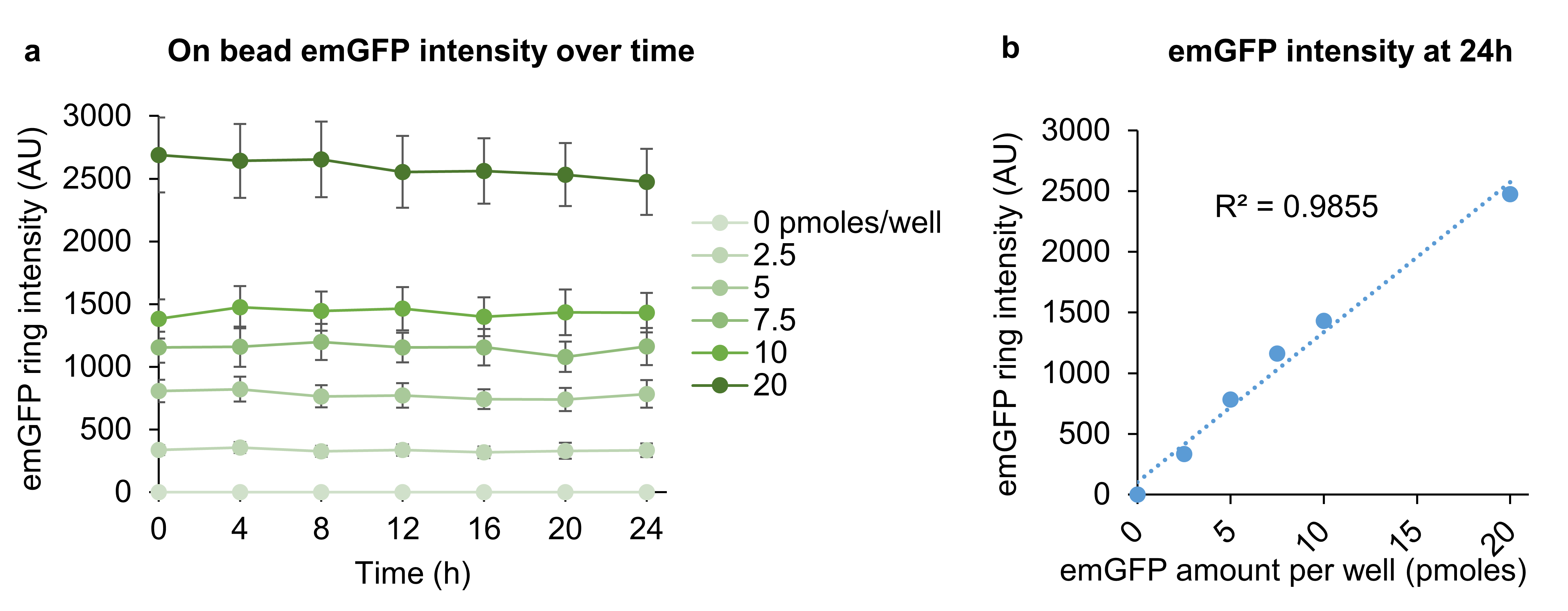
*

**Figure S7: Ring fluorescence intensity is stable over time and remains linearly proportional to the amount of bead-bound fluorescent substrate.**

(**a**) His6-tagged emGFP-Ube2C fusion in different amounts (0-20 pmoles per well) was bound to Ni^2+^NTA agarose beads as described in the Methods. After washes, the emGFP beads were placed in a 384-well plate and imaged using the Opera^TM^ microscope (Perkin Elmer). The emGFP fluorescence intensity was averaged for each well. Detected emGFP intensity remained stable over 24 h of imaging for amounts up to 10 pmoles/well and was proportional to the amount of the bead-bound emGFP (**b**).

| **Reaction** | **On bead (His6-tagged)** | **In solution (besides Ub/ULM)** | **Modifier type** |
| --- | --- | --- | --- |
| **E1 loading with ubiquitin** | Ube1 |  | FITC-Ub, Cy5-Ub, TMR-Ub |
| **E2 charging with ubiquitin** | Ube2C, Ube2D1, Ube2D2, Ube2D4, Ube2E2, Ube2G1, Ube2G2, Ube2H, Ube2K, Ube2L3, Ube2N, Ube2R1, Ube2R2, Ube2S, Ube2U, Ube2W | Ube1 | FITC-Ub or Cy5-Ub |
| **E2 charging with NEDD8** | Ube2M | Uba3/NAE1 | FITC-NEDD8 |
| **Ratiometric E2 charging with ubiquitin** | emGFP or mTurq2 fusions: Ube2C, Ube2D1, Ube2D4, Ube2E2, Ube2G1, Ube2G2, Ube2H, Ube2K, Ube2L3, Ube2L6, Ube2Q2, Ube2R1, Ube2R2, Ube2S, Ube2W | Ube1 | Cy5-Ub |
| **HECT E3 charging/autoubiqutination** | E6AP, CHIP | Ube1, Ube2L3 | Cy5-Ub |
| **Substrate ubiquitination** | p53 | Ube1, Ube2D3, Mdm2 | FITC-Ub |
| **2-step reaction: ubiquitin activation and transfer to E2** | Ube1 | Ube2R1, Ube2L3 | FITC-Ub, Cy5-Ub |
| **3-step reaction: ubiquitin activation, transfer to an E2 and transfer to a HECT E3** | Ube1 and E6AP | Ube2L3 | Cy5-Ub |
| **Ubiquitin binding** | Ube2R1 | CC0651 | Cy5-Ub |
| **Cross-pathway reactivity** | Ube2R1 or Ube2M | Ube1, Uba3/NAE1 | Cy5-Ub, FITC-NEDD8 |
| **Chemical modulators of ubiquitin-like reactions** | Ube1 | PYR-41 | FITC-Ub |
|  | Ube2M | Uba3/NAE1, MLN4924 | FITC-NEDD8 |
|  | Ube2R1 | CC0651 | Cy5-Ub |
|  | Various E2s | Ube1, BAY 11-7082, CC0651 | Cy5-Ub |
|  | p53 | Ube1, Ube2D3, Mdm2, nutlin-3 | FITC-Ub |
| **Screening for chemical modulators** | Ube2C | Ube1, >2000 compounds | Cy5-Ub |
| **SAR** | Ube2C | Ube1, ~100 analogues | Cy5-Ub |
| **Evaluation of compound specificity** | Ube2C, other E2s; Ube1 | Ube1, hit compounds | Cy5-Ub |
|  | Various E2s | Ube1, BAY 11-7082, CC0651 | Cy5-Ub |
|  | Ube1 and E6AP | Ube2L3, PYR-41, BAY 11-7082, clomipramine, heclin | Cy5-Ub |
| **Evaluation of activity of a new chemical probe** | Ube1, Ube2L3 | Ube1, Ube2L3, probe | Cy5-Ub |

**Table S1: UPS-CONA is a broadly applicable assay.**

Listed are different ubiquitin-related reactions which have been tested using UPS-CONA. Various enzymes/substrates were immobilized on bead to monitor conjugation of Ub or ULM with fluorescent labels including Cy5, TMR and FITC. Reactions also included testing inhibitors against ubiquitination enzymes.
